# Does serum calcium level play a practical role in calciphylaxis in hemodialysis patients?

**DOI:** 10.1080/0886022X.2021.1999828

**Published:** 2021-11-09

**Authors:** Lijing Zhang, Yu Zhao, Zhong Guo, Jianxiu Ma, Yanqing Ma, Zhanli Yang

**Affiliations:** Department of Medicine, Northwest Minzu University, Lanzhou, PR China

Dear Editor,

We read with interest Dr. Liu and his colleagues’ [1] recent article published in Renal Failure: ‘Risk factors for calciphylaxis in Chinese hemodialysis patients: a matched case-control study’. Calcific uremic arteriolopathy (CUA), also called calciphylaxis, is a rare but devastating disease predominantly affecting patients with end-stage kidney disease (ESKD) on dialysis. The exact incidence and prevalence are unknown. The article enrolled 20 hemodialysis patients with newly diagnosed calciphylaxis and 40 noncalciphylaxis patients with the same age and duration of dialysis as control group for a retrospective matched case-control study. The univariate regression analysis indicated that corrected serum calcium level were significantly associated with calciphylaxis (OR 4.498, *p* = 0.023), but multivariate logistic analysis showed that it was not an important risk factor. What is more, the laboratory examination results showed that there were significant differences in corrected serum calcium (2.46 ± 0.28 vs 2.30 ± 0.20, *p* = 0.015), no significant differences in serum calcium (2.36 ± 0.27 vs 2.28 ± 0.19, *p* = 0.221). However, as mentioned in the article ‘the levels of serum calcium, serum phosphate and serum ALP in calciphylaxis group were significantly increased’. This contradiction made me pay special attention because it caused me some confusion.

Firstly, a matched case-control study conducted by Nigwekar et al. [2] 62 adult maintenance hemodialysis (HD) patients with biopsy-confirmed calciphylaxis and 124 controls. Serum calcium (both uncorrected and albumin corrected; *p* = 0.03) were higher for cases compared to controls. In unadjusted and adjusted logistic regression analysis, corrected serum calcium was positively associated with calciphylaxis as mentioned in table 2 and table 3 (OR 1.83, 95% CI 1.13–2.98, *p* = 0.02; OR 2.25, 95% CI 1.14–4.43, *p* = 0.02). Furthermore, uncorrected serum calcium was also positively associated with calciphylaxis in the above two models (OR 1.93, 95% CI 1.15–3.24; OR 2.23, 95% CI 1.10–4.21).

Secondly, Nigwekar et al. [3] used the data from a large dialysis organization to investigate whether baseline factors recorded at hemodialysis initiation would identify patients at risk for future calciphylaxis in a matched case-control study. The article includes 1030 HD patients with newly diagnosed calciphylaxis and 2060 matched controls. It showed that higher albumin-corrected serum calcium (for every 1 mg/dl increase: OR, 1.33; 95% CI, 1.12–1.61) were associated with increased odds of future calciphylaxis development in multivariable conditional logistic regression analysis. However, uncorrected serum calcium was not included in the above multivariate logistic regression analysis.

Finally, Gaisne et al. [4] conducted a retrospective cohort study on CUA cases identified in western France. 89 CUA (calciphylaxis) cases were identified, including 19 non dialyzed and 70 dialyzed patients. They measured the laboratory parameters at onset of calciphylaxis in patients and paired dialysis controls. It showed that both uncorrected and albumin corrected serum calcium were higher than controls (*p* = 0.04 and *p* < 0.001). Interestingly, the results of univariate and multivariate logistic regression analysis were the same as those of Dr. Liu and his colleagues. And similar findings were reported by Hayashi et al. [5].

Based on the above evidence, we wonder whether higher serum calcium is a risk factor of calciphylaxis? And how does the patient's serum calcium change when calciphylaxis is diagnosed? Many studies suggest that decreased level of serum albumin is a vital high-risk factor for calciphylaxis. So we speculate that hypoproteinemia is one of the factors that may mediate the differences between serum calcium and corrected serum calcium. Thus, we should pay more attention to the level of corrected serum calcium when serum albumin decrease in calciphylaxis. As we all know, accurate identification of risk factors for calciphylaxis is necessary to develop preventive strategies for this morbid disease. So, further prospective studies of larger sample size are needed to confirm the relationship between serum calcium and calciphylaxis.
